# Picture quiz

**Published:** 2018-06-03

**Figure:**
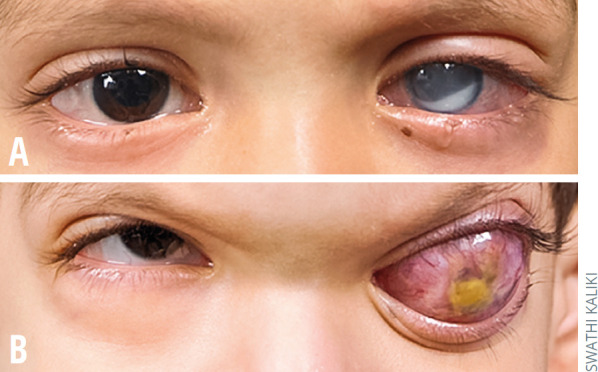


A 2-year-old male is brought by his parents. The history is that a white reflex was noted in the eye several months ago. The eye has now become red and painful (**A**). The patient is then lost to follow-up and comes back 6 months later with proptosis and frontal bossing (**B**).

**Tick ALL that are TRUE**

**Question 1 What is your diagnosis?**
  □ **a.** Endophthalmitis
  □ **b.** Coats disease
  □ **c.** Congenital glaucoma
  □ **d.** Retinoblastoma
  □ **e.** Staphyloma
**Question 2 How will you confirm the extent of disease?**
  □ **a.** Clinical examination
  □ **b.** Computed tomography or magnetic resonance imaging of the orbit
  □ **c.** Cytology of bone marrow aspirate
  □ **d.** Cytology of cerebrospinal fluid
  □ **e.** All the above
**Question 3 What is the treatment of choice for the left eye?**
  □ **a.** Primary enucleation followed by 6 cycles of systemic chemotherapy
  □ **b.** Primary exenteration followed by 6 cycles of systemic chemotherapy
  □ **c.** Primary enucleation followed by orbital external beam radiotherapy
  □ **d.** Primary exenteration followed by orbital external beam radiotherapy
  □ **e.** Combination treatment of systemic chemotherapy, enucleation, and orbital external beam radiotherapy

## ANSWERS

(d) Retinoblastoma
(e) All the above. Clinical examination confirms the laterality of disease. Imaging of the orbit confirms the presence of extraocular extension, and confirms the presence or absence of optic nerve extension with/without associated intracranial extension. The two most common sites of metastases from retinoblastoma are cerebrospinal fluid and bone marrow. Cytology from these two sites is indicated in all cases of retinoblastoma with extraocular extension.
(e) Combination treatment of systemic chemotherapy, enucleation, and orbital external beam radiotherapy. Primary enucleation or exenteration should not be done in cases with overt extraocular or optic nerve tumour extension, since this can worsen the prognosis due to tumour spill over. The ideal treatment modality is a multimodal approach of chemoreduction followed by surgery, radiotherapy, and adjuvant chemotherapy.

